# Continuous positive airway pressure for bronchiolitis in a general paediatric ward; a feasibility study

**DOI:** 10.1186/1471-2431-14-122

**Published:** 2014-05-12

**Authors:** Knut Øymar, Kjersti Bårdsen

**Affiliations:** 1 Department of Paediatrics, Stavanger University Hospital, PO box 8100, 4068 Stavanger, Norway; 2 Department of Clinical Science, University of Bergen, Bergen, Norway

**Keywords:** Bronchiolitis, CPAP, Infant, Intensive care unit, RSV, Ward

## Abstract

**Background:**

Continuous positive airway pressure (CPAP) is commonly used to relieve respiratory distress in infants with bronchiolitis, but has mostly been studied in an intensive care setting. Our prime aim was to evaluate the feasibility of CPAP for infants with bronchiolitis in a general paediatric ward, and secondary to assess capillary PCO_2_ (cPCO_2_) levels before and during treatment.

**Methods:**

From May 1^st^ 2008 to April 30^th^ 2012, infants with bronchiolitis at Stavanger University Hospital were treated with CPAP in a general paediatric ward, but could be referred to an intensive care unit (ICU) when needed, according to in-house guidelines. Levels of cPCO_2_ were prospectively registered before the start of CPAP and at approximately 4, 12, 24 and 48 hours of treatment as long as CPAP was given. We had a continuous updating program for the nurses and physicians caring for the infants with CPAP. The study was population based.

**Results:**

672 infants (3.4%) were hospitalized with bronchiolitis. CPAP was initiated in 53 infants (0.3%; 7.9% of infants with bronchiolitis), and was well tolerated in all but three infants. 46 infants were included in the study, the majority of these (n = 33) were treated in the general ward only. These infants had lower cPCO_2_ before treatment (8.0; 7.7, 8.6)(median; quartiles) than those treated at the ICU (n = 13) (9.3;8.5, 9.9) (p < 0.001). The level of cPCO_2_ was significantly reduced after 4 h in both groups; 1.1 kPa (paediatric ward) (p < 0.001) and 1.3 kPa (ICU) (p = 0.002). Two infants on the ICU did not respond to CPAP (increasing cPCO_2_ and severe apnoe) and were given mechanical ventilation, otherwise no side effects were observed in either group treated with CPAP.

**Conclusion:**

Treatment with CPAP for infants with bronchiolitis may be feasible in a general paediatric ward, providing sufficient staffing and training, and the possibility of referral to an ICU when needed.

## Background

Bronchiolitis is one of the most common reasons for hospitalisation in infants [1-3]. Symptoms may include coughing, wheezing, eating difficulties and apnoea. Bronchiolitis is commonly caused by respiratory syncytial virus (RSV) occurring in epidemics during the winter season, but other viruses may be involved [4]. Treatment is mainly supportive, with oxygen, fluid therapy and respiratory support when needed [1-3]. Inhalations with (racemic) adrenaline/epinephrine are commonly used in some countries, but the evidence is sparse [5]. Recently, studies of inhalations with hypertonic saline have been promising, but more studies are awaited [6].

Patients at risk of severe bronchiolitis include infants < 3 months of age, those with chronic lung disease after prematurity, other chronic lung diseases, congenital heart disease and neuromuscular impairment [1-3,7]. In severe bronchiolitis, respiratory failure may develop in spite of standard treatment; symptoms may then include apnoea, hypoxia and respiratory distress [7]. Low oxygen saturation, high oxygen requirement and increasing levels of CO_2_ (arterial or capillary) may indicate the need for ventilatory support [8]. The decision to intubate and mechanically ventilate an infant with bronchiolitis is based on a combination of clinical signs and laboratory results [8,9].

First described in 1981 [10], continuous positive airway pressure (CPAP) has been given to children with severe bronchiolitis in order to support ventilation and avoid the need for mechanical ventilation [8]. CPAP works by keeping airways open, increasing clearance of secretion, improving gas exchange and reducing the work of breathing [11,12]. Two small randomised studies have demonstrated the effect of CPAP on reducing capillary PCO_2_ and clinical scores [13,14]. Otherwise, only observational studies has been published [10,11,15-19], and no study has demonstrated that the use of CPAP reduces the need for mechanical ventilation in children with severe bronchiolitis [7,8].

In the published studies, the infants with bronchiolitis were referred to an intensive care unit (ICU) [11,14-18] or high dependency unit [19] when treated with CPAP. Treatment in an ICU is resource demanding and may be frightening for the parents. Treatment with CPAP in general paediatric wards could possibly be easier and less resource demanding; with a potentially lower threshold to initiate treatment. At the paediatric department Stavanger University Hospital we have during the four seasons from May 1^st^ 2008 to April 30^th^ 2012 treated children with bronchiolitis with a simple CPAP setup suitable for general paediatric wards, with possible transfer to an ICU when needed. In this article we present the results and experience with this method in a population based setting.

## Methods

### Setting and patients

Stavanger University Hospital is both a local and secondary referral hospital and the only hospital for children in South Rogaland, Norway. The annual number of births is approximately 5000 (2011). During the study period, the paediatric ward for children with infectious diseases had 11 beds in single-bed rooms. The ward was staffed with eight nurses during daytime, six during weekends and evenings and four during nights. Three physicians attended the ward during daytime, and two were available during weekends, evenings and nights.

All nurses and physicians in the ward involved with CPAP therapy were trained in practical and technical details before being allowed to participate. An annual training program before each bronchiolitis season was established. Detailed written practical and technical procedures were available for both the physicians and nurses.

The hospital further had a seven-bed ICU for patients of all ages after the neonatal period, which also admitted infants with bronchiolitis in need of intensive care. Referral from the paediatric ward to the ICU could be organised within a few minutes if necessary.

Bronchiolitis was defined as an acute respiratory infection in a child < 12 months of age with typical symptoms of wheezing (prolonged expiration) [1-3]. Infants with bronchiolitis needing hospitalisation were referred to the paediatric ward for infectious diseases. Nasopharyngeal mucus was examined for RSV by direct immunofluorescence in all patients (bioMe’rieux, Marcyl’E’toile, France).

The standard treatment for bronchiolitis in the department is oxygen when needed (to keep the oxygen saturation ≥ 92-94%), fluid and nutritional support (by nasogastric tube or intravenously). During the study period, we regularly treated the infants with racemic adrenaline if the child had bronchopulmonary obstruction or severe cough; 2–4 mg in 2 ml isotonic saline every 2–4 hours as needed [5], or with only isotonic saline when needed.

Traditionally, when an infant with bronchiolitis demonstrate signs of respiratory failure we have referred the infant to the ICU for treatment with CPAP or mechanical ventilation. From 2008 we have aimed to initially treat infants in need of CPAP in the general paediatric ward without initial referral to the ICU. The study period included four years from May 1^st^ 2008 to April 30^th^ 2012. Only children living in the area for Stavanger University Hospital were included, making the study population based.

### CPAP – indications and setup

Children treated with CPAP were given oxygen, fluid support and nebulised racemic adrenaline in advance. The decision to start treatment with CPAP was taken by the physician responsible, but according to in-house guidelines (see list of criteria below).

List of criteria for starting treatment with continuous positive airway pressure (CPAP) or referral to intensive care unit (ICU) in infants with bronchiolitis at Stavanger University Hospital

1. CPAP is considered for a child with bronchiolitis with

a. Recurrent episodes of apnoea

b. Severe respiratory distress; retractions, severe wheezing

c. Increasing oxygen supply in spite of other treatment

d. Increasing capillary CO_2_ (> 6.5-7.0 kPa)

e. Young age (<2-3 months)

2. Referral to the ICU is considered when an infant with bronchiolitis in spite of treatment with CPAP at the paediatric ward have

a. Severe respiratory distress (severe retractions, tachypnoe > 70/min)

b. High or increasing capillary CO_2_ (> 6.5-7.0 kPa)

c. Severe apnoea

d. Poor general condition or discomfort

e. Unstable circulation

In the paediatric ward we used the CPAP GoodKnight 420E® (Puritan Bennett, Coviden, Mansfield, MS, USA) with two different nasal masks with leaks; ProfileLite Small Child (Philips Respironics, Tangmere, UK) or Infant Bubble Mask (Sullivan Infant Bubble Mask, Resmed, San Diego, CA, USA) which were individually adapted.

The CPAP pressure was set at 5 cm H_2_O in all cases. Extra oxygen was given as 100% oxygen into the circuit as needed to keep the SpO_2_ within excepted limits. The nebuliser Aeroneb ProX (Aerogen, Galway, Ireland) was connected to the circuit, allowing inhalations without changing the gas flow to the patient, otherwise the gas was not humidified. Careful nasal suctioning was performed in infants with copious secretion.

For infants treated with CPAP in the ward, SpO_2_ was continuously monitored by pulse oximetry. A nurse was permanently in the room during the initial phase, during weaning by frequent observations. One of the parents was always with the child in the room. A physician could attend immediately if necessary.

Referral to the ICU was considered if the child was not successfully treated at the paediatric ward; according to criteria given above. Infants referred to the ICU were treated with nasal CPAP using the Dräger Evita XL ventilator (Dräger Medical, Lübeck, Germany) with nasal prongs (Fischer & Paykel Healthcare, Irvine, CA). Initial CPAP pressure was set to 5 cm H_2_O.

We aimed at measuring a capillary PCO_2_ (cPCO_2_) before starting treatment with CPAP, 4–6 hours after CPAP had been initiated, and approximately 12, 24 and 48 hours after the start of treatment if the infant was still on CPAP. Samples for capillary PCO_2_ was taken and analyzed by laboratorial staff. Data were prospectively registered by nurses on a special record for the project, missing data were retrospectively collected from hospital records. Arterial blood gases were not measured and respiratory distress not systematically registered.

We considered the procedure described as the best treatment for bronchiolitis available based on the literature, and no control group was included [1-3,7]. The procedure was therefore not considered as a research protocol; the regional ethical committee was consulted and waived the need for approval.

### Statistics

Comparisons between groups were analysed by non-parametric tests for variance; the Kruskal-Wallis test for independent samples and Friedman test for related samples. A p-value < 0.05 was regarded as statistically significant, and all analyses were two-tailed. Data were analysed using the SPSS version 18.0 statistical package (SPSS, Chicago, IL, USA).

## Results

**Figure 1:**
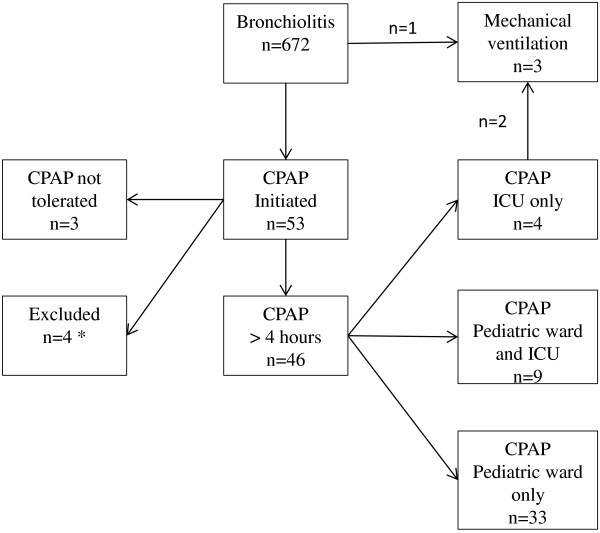
**Overview of infants < 12 months of age hospitalized for bronchiolitis during four years at Stavanger University Hospital and the number of infants given continuous positive airway pressure (CPAP) or mechanical ventilation.** Infants were either treated in a general paediatric ward or an intensive care unit (ICU).* Four children excluded from the study due to chronic disease.

During the four winter seasons, a total number of 672 infants younger than 12 months of age were hospitalised for bronchiolitis; 3.4% of all infants < one year of age in the catchment area. Of these, 339 tested positive for RSV (50%). In total, treatment with CPAP was initiated in 53 infants with bronchiolitis during the four seasons; 0.3% of all infants < one year of age and 7.9% of all children hospitalized for bronchiolitis. Three infants were given mechanical ventilation for bronchiolitis during the period (0.4%); one of these (age 10 months) was ventilated from admission without initial CPAP due to rapid and severe clinical deterioration. Two infants failed on CPAP and were given mechanical ventilation, one due to high cPCO_2_ and respiratory distress and one infant due to severe apnoea in spite of normal cPCO_2_ (Figure 1). For three infants, treatment with CPAP were initiated, but was unsuccessful due to non-cooperating child. Four children were excluded from analyses due to other diseases or treatment with CPAP < four hours (Figure 1).

Among the remaining 46 infants, 33 were treated with CPAP only in the general paediatric ward. Thirteen were referred to the ICU, for nine of those due to respiratory distress in spite of CPAP given at the ward. Four children were given treatment only in the ICU due to severe respiratory distress at admission (Figure 1).

When analysing data, children treated only in the paediatric ward and those treated in the ICU were compared. Clinical characteristics and levels of cPCO_2_ before and after four hours treatment with CPAP for both groups are given in Table 1. Children treated in the ICU had a higher cPCO_2_ before treatment and after 4 hours treatment with CPAP compared to those only treated in the paediatric ward, but no other significant differences were observed between the groups.

**Figure 2:**
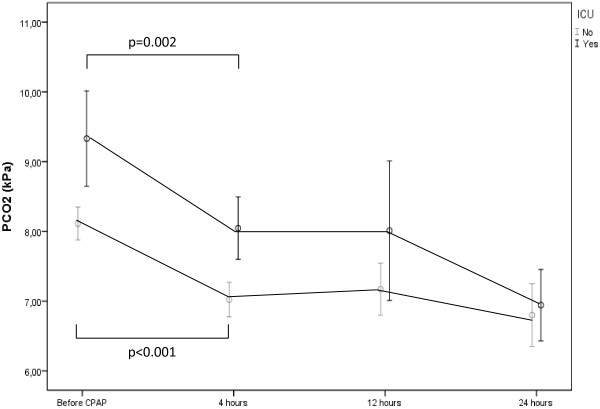
**Capillary PCO**_**2 **_**before the start of treatment with continuous positive airway pressure (CPAP) and 4, 12 and 24 hours after the start of treatment in infants with bronchiolitis during four years at Stavanger University Hospital.** Infants were either treated in a ordinary paediatric ward only ward (lower line) or an intensive care unit (ICU) (upper line).

The levels of cPCO_2_ before CPAP was given (n = 33/13) and after 4 hours (n = 33/13), 12 hours (n = 21/10), and 24 hours (n = 14/7) for both groups are demonstrated in Figure 2. For both groups there were a reduction in in cPCO_2_ after four hours treatment, the reduction of the median cPCO_2_ was 1.1 (paediatric ward only) and 1.3 kPa (ICU) (p < 0.001 and p = 0.002) (Figure 2). In one child, the cPCO_2_ increased from 8.4 kPa to 9.2 kPa 4 hours after CPAP was initiated, but declined to 7.4 kPa the next 12 hours. Increasing cPCO_2_ was not observed in any other infants after treatment with CPAP was initiated.

**Table 1:** Clinical and laboratory characteristics in infants with bronchiolitis treated with continuous positive airway pressure (CPAP) at Stavanger University Hospital during four years

	**Paediatric ward N = 33**	**ICU n = 13**	**p-value**
Gender (boys/girls)	17/16	10/3	0.184
Gestational age (weeks)	38 (34, 40)	37 (34, 38)	0.383
Age at admission (weeks)	34 (20, 61)	25 (18, 40)	0.157
RS-virus (yes/no)	26/7	11/2	0.712
cPCO_2_ before CPAP	8.0 (7.7, 8.6)	9.3 (8.5, 9.9)	<0.001
cPCO_2_ 4 hours	6.9 (6.6, 7.4)	8.0 (7.5, 8.5)	<0.001
Duration of CPAP (hours)	24 (8, 44)	30 (19, 60)	0.195
Length of total hospital stay	7 (5, 8)	8 (6, 11)	0.085

Children in the first group were treated in the paediatric ward only, and children in the second group in the intensive care unit (ICU) only (n = 4) or referred to the ICU after treatment in the paediatric ward (n = 9). Results are given as median (quartiles).

The CPAP was generally well tolerated. Except for the three infants not cooperating, no significant side effects were observed.

## Discussion

The results of this study suggest that CPAP for infants with bronchiolitis may be feasible in a general paediatric ward for the majority of infants. We showed a significant decline in median cPCO_2_ four hours after the initiation of treatment also in this setting, the majority of infants tolerated CPAP well, and no significant side effects were observed. However, sufficient and trained staffing and the possibility of referral to an ICU should be a prerequisite for CPAP therapy in general wards.

Our study may be the first to show the results of treatment with CPAP in a general paediatric ward. In the majority of children, referral to a higher level of care was not necessary, and the effect of treatment measured by cPCO_2_ was comparable to those treated at the ICU and to the results in other studies. Treatment in general wards may have economic benefits, and be more convenient for the parents [20]. However, we consider it mandatory that sufficient staffing (nurses and physicians) are available, and that sufficient training is provided. Moreover, as almost one third of the children were in need of referral to an ICU, the possibility for such referral must be easily available. Except for one infant with severe apnoea in need of mechanical ventilation, significant apnoea was not observed in any of the infants treated with CPAP. This may however be more common than observed by us, and may be a reason for referral to and ICU.

The observed effect of CPAP on cPCO_2_ in our study is in agreement with previous studies. The effect of CPAP in bronchiolitis has so far been studied in only two small randomized studies. In a blinded randomised cross-over study by Thia et al., 31 children with bronchiolitis and a capillary cPCO_2_ > 6 kPa were given either standard treatment with or without CPAP for 12 hours. For those given CPAP first, the mean reduction in cPCO_2_ was 1.35 kPa, which was more than for those given CPAP after 12 hours [13]. Recently, Milesi et al. demonstrated that in 10 children with severe bronchiolitis, CPAP was effective in decreasing respiratory work compared to the 9 controls [14]. Both these studies emphasized the possible importance of early introduction of CPAP in children with severe bronchiolitis.

The other published studies have been observational with a before-after design. Only one study has been performed outside an ICU; Lazner et al. recently published a retrospective study with from a high dependency unit [19]. They found that different methods of non-invasive ventilation, mainly CPAP with a preset-level, were effective in 80% of infants receiving this support. A beneficial effect was seen both on the respiratory work, oxygen saturation and cPCO_2_. The average levels of cPCO_2_ were 8.5 kPA before treatment and 7.3 kPa after 4 hours in responders, in accordance with our results.

We did not aim to compare the efficacy of CPAP between an ICU and general paediatric ward, as the infants with more severe symptoms were referred to the ICU. However, those referred to the ICU had higher levels of cPCO_2_, suggesting that that this criterion may help deciding which patients that can be managed in general wards.

As in our study, few complications have been described for infants treated with CPAP [11,14-16,19], and the fear of side effects such as increasing cPCO_2_ or respiratory distress when starting CPAP, does not seem to be an argument against giving CPAP at a low level of care. Giving CPAP in general wards may increase the availability for this treatment, and open for the early introduction of CPAP in infants with more moderate symptoms. A recent study comparing to periods with different strategies, suggests that a pre-emptive use of CPAP in bronchiolitis may improve both clinical and economic outcomes in infants with bronchiolitis [20].

The treatment with CPAP in our study included the use of a nasal mask, and a continuous pressure of 5 cm H_2_O. Nasal prongs have been used successfully in other studies [21], and the choice of delivery system may not be essential for the effect of the treatment. We chose a CPAP apparatus produced for home ventilation due to the simplicity of handling and because compressed air was not needed. Recently, Essouri et al. also suggested that a CPAP level of 7 cm H_2_O may have the best effect on respiratory efforts in infants with bronchiolitis [12].

As the present study was population based and continued for four whole years, it also adds epidemiological based data regarding the number of children with bronchiolitis needing hospitalization, and how many will be in need of ventilatory support. However, both these variables may depend on local criteria for treatment. The hospitalization rate observed by us may be slightly higher than that observed in other studies both in Norway [22] and other countries [2]. This may possibly be explained by an increasing rate of hospitalization for bronchiolitis which has been observed [23]. We have not identified other population based studies describing the incidence of CPAP in children with bronchiolitis below one year of age.

The population based design is a positive feature of this study, as there was no selection of children on any level. A weakness is that only cPCO_2_ was used as an objective parameter of efficacy, and not respiratory work or oxygen saturation. Relative few patients were included in this single centre study, and larger studies including more clinical parameters could be initiated to evaluate the safety of giving CPAP in general wards.

In a recent review, Donlan et al. concluded that in spite of the increasing use of CPAP in bronchiolitis, evidence supporting the use of CPAP to reduce PCO_2_ and respiratory distress in bronchiolitis is of low quality, and that there is no evidence that CPAP reduces the need for intubation [8]. Consequently, there is still need for high quality randomized studies of the use of nasal CPAP in children with bronchiolitis, not only to study the effect of CPAP per se, but also to see if the early introduction of CPAP in children with less severe symptoms may improve the outcome or the need for other support such as mechanical ventilation.

## Conclusion

Our results suggest that for infants with bronchiolitis in need of treatment with CPAP, this therapy may be feasible in a general paediatric ward for the majority of infants, provided sufficient staff, training and monitoring. This may have economic benefits, be preferable for parents and permit for more infants to be treated with CPAP for this common disease.

## Competing interests

The authors declare that they have no competing interests.

## Authors’ contributions

KØ planned the study, analysed data and wrote a draft of and completed the manuscript. KB contributed to the planning of the study, was responsible for collection of data and approved the final manuscript.

## Pre-publication history

The pre-publication history for this paper can be accessed here:

http://www.biomedcentral.com/1471-2431/14/122/prepub
